# OsWRKY114 Inhibits ABA-Induced Susceptibility to *Xanthomonas oryzae* pv. *oryzae* in Rice

**DOI:** 10.3390/ijms23158825

**Published:** 2022-08-08

**Authors:** Seungmin Son, Jong Hee Im, Giha Song, Suhyeon Nam, Sang Ryeol Park

**Affiliations:** 1 National Institute of Agricultural Sciences, Rural Development Administration, Jeonju 54874, Korea; 2 Department of Horticulture, Michigan State University, East Lansing, MI 48824, USA; 3 Department of Crop Science & Biotechnology, Jeonbuk National University, Jeonju 54896, Korea

**Keywords:** abscisic acid, bacterial blight, innate immunity, OsWRKY114, rice, *Xanthomonas oryzae* pv. *oryzae*

## Abstract

The phytohormone abscisic acid (ABA) regulates various aspects of plant growth, development, and stress responses. ABA suppresses innate immunity to *Xanthomonas oryzae* pv. *oryzae* (*Xoo*) in rice (*Oryza sativa*), but the identity of the underlying regulator is unknown. In this study, we revealed that OsWRKY114 is involved in the ABA response during *Xoo* infection. ABA-induced susceptibility to *Xoo* was reduced in *OsWRKY114*-overexpressing rice plants. OsWRKY114 attenuated the negative effect of ABA on salicylic acid-dependent immunity. Furthermore, OsWRKY114 decreased the transcript levels of ABA-associated genes involved in ABA response and biosynthesis. Moreover, the endogenous ABA level was lower in *OsWRKY114*-overexpressing plants than in the wild-type plants after *Xoo* inoculation. Taken together, our results suggest that OsWRKY114 is a negative regulator of ABA that confers susceptibility to *Xoo* in rice.

## 1. Introduction

Phytohormones, such as abscisic acid (ABA), salicylic acid (SA), jasmonic acid (JA), ethylene (ET), auxins, brassinosteroids, cytokinins, and gibberellins, play critical roles in various plant processes during growth, development, and stress responses [1,2,3]. ABA is a major phytohormone that regulates embryo maturation, seed dormancy, germination, flowering, and senescence [4]. Moreover, ABA is a key phytohormone that modulates various stress responses in plants [5,6]. Endogenous ABA contents increase in response to abiotic stress (e.g., drought and salt), making ABA an important factor in a plant’s ability to overcome harsh environmental conditions [7,8]. ABA is also involved in biotic stress responses: ABA inhibits pathogen entry by inducing stomatal closure and increases plant disease resistance [9,10,11]. However, ABA commonly suppresses the major biotic stress-related phytohormone SA and is thus a negative regulator of plant innate immunity [12]. Therefore, ABA can increase the susceptibility of a wide variety of pathogens [13,14,15,16,17].

Indeed, exogenous application of ABA or the accumulation of endogenous ABA causes an enhanced susceptibility to bacterial and fungal pathogens in various plants [18,19,20,21,22,23]. For example, exogenous ABA increases the susceptibility of biotrophic bacterial pathogen *Pseudomonas syringae* pv. *tomato* (*Pst*) DC3000, and the bacterial effector AvrPtoB induces the expression of gene encoding 9-*cis*-epoxycarotenoid dioxygenase 3 (NCED3), a key enzyme in pathogen-induced ABA biosynthesis, to suppress plant defense response in Arabidopsis (*Arabidopsis thaliana*) [24]. Furthermore, ABA and the overexpression of *NCED* genes (i.e., *NCED3* and *NCED5*) increase the growth of the *P*. *syringae* strains in Arabidopsis [25].

The crosstalk between ABA and three major defense phytohormones, such as SA, JA, and ET, is important for plant resistance during disease development. ABA has an antagonistic interaction with SA signaling that confers resistance to biotrophic pathogens, while it can act antagonistically or synergically with JA/ET signaling involved in resistance to necrotrophic pathogens [5,26,27]. Therefore, ABA-mediated SA suppression plays a critical role in the susceptibility of biotrophic pathogens. In Arabidopsis, ABA decreases the expression of *isochorismate synthase 1* (*ICS1*), a key enzyme in SA biosynthesis, and plays a central role in the attenuation of the SA-mediated plant defense mechanism against *Pst* DC3000 [28]. ABA and NCEDs increase the susceptibility of the *P*. *syringae* strains through antagonistic relationship with SA, while ABA promotes JA synthesis [25]. The pyrabactin-resistance 1/pyrabactin-resistance-like/regulatory component of the ABA receptor (PYR/PYL/RCAR), such as PYR1, compromises SA defense signaling against *Pst* DC3000, while it enhances ET signaling [27]. In rice (*Oryza sativa*), ABA also increases the susceptibility of the biotrophic bacterial pathogen *Xanthomonas oryzae* pv. *oryzae* (*Xoo*) and inhibits SA-mediated defense response [29]. However, the signaling components that regulate ABA-induced pathogen susceptibility remain largely elusive.

The plant-specific WRKY transcription factor family plays substantial roles in plant growth, development, and biotic/abiotic stress responses [30]. WRKY transcription factors comprising a large number of members are classified by one or two 60-amino acid WRKY domains (WDs), which consist highly conserved WRKY motif [WRKYGQK] and zinc finger-like motif [C_2_H_2_ or C_2_HC] (i.e., Group I, two WDs; Group II, one WD with C_2_H_2_ zinc finger; and Group III, one WD with C_2_HC zinc finger) [31]. They directly bind to W-box motif (TTGACC/T) in promoter regions of target genes and regulate the expression of them [30]. WRKYs modulate various signaling cascades as either activators or repressors [31,32,33,34,35]. In rice, there are 103 genes encoding WRKY transcription factors, including 28 Group III members [36,37]. Recent studies have indicated that several rice WRKYs are associated with ABA. For instance, OsWRKY5 increases ABA-induced leaf senescence by upregulating ABA biosynthetic genes and raising endogenous ABA level [38]. OsWRKY29 negatively regulates ABA signaling and decreases the expression of several ABA-related genes during seed dormancy [39]. OsWRKY50 inhibits ABA-dependent seed germination and seedling growth, while enhancing salt stress tolerance via an ABA-independent pathway [40]. However, whether any OsWRKYs regulate ABA signaling during biotic stress is unknown. We previously revealed that OsWRKY114 enhances innate immunity to *Xoo* through the direct upregulation of pathogenesis-related (*PR*) genes, such as *OsPR1a* and *chitinase* [41]. Moreover, most recently, it was reported that OsWRKY114 decreases the expressions of *PYR/PYL/RCAR* genes that improve drought tolerance through stomatal closure [42]. However, the biological function of OsWRKY114 in ABA-pathogenesis has yet to be elucidated. Here, we established a molecular mechanism for how OsWRKY114 inhibits ABA-mediated susceptibility in *Xoo* infection.

## 2. Results

### 2.1. OsWRKY114 Expression Is Modulated by ABA

**Figure 1:**
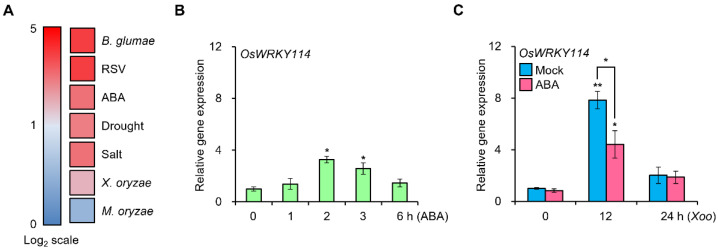
ABA modulates the transcription of *OsWRKY114* during *Xoo* inoculation. (**A**) Expression profile of *OsWRKY114* under various stress conditions. Gene expression data were obtained from Genevestigator. (**B**) Relative *OsWRKY114* transcript level after ABA treatment, as determined by RT-qPCR. Four-week-old rice plants were sprayed with 100 µM ABA and incubated for the indicated times. cDNA was synthesized from total RNA in rice leaves. *OsActin* was used as an internal control. Data are shown as means ± standard deviation (SD). * *p* < 0.05 by *t*-test relative to the 0 h sample. (**C**) Relative *OsWRKY114* transcript level in ABA-treated plants after *Xanthomonas oryzae* pv. *oryzae* inoculation. Four-week-old rice plants were pretreated with 100 µM ABA or mock (0.2% [*v*/*v*] Tween 20) solution. Three days later, leaves were inoculated with *Xoo* and collected after the indicated incubation times. cDNA was synthesized from total RNA in rice leaves. *OsActin* was used as an internal control. Data are shown as means ± SD. * *p* < 0.05, ** *p* < 0.01, determined by *t*-test relative to the 0 h mock-treated sample and between the indicated comparing samples.

To explore the expression patterns of *OsWRKY114*, we analyzed a public expression database of Genevestigator. *OsWRKY114* expression is affected by various biotic/abiotic stresses and ABA (Figure 1A). Since ABA is an important phytohormone involved in various stress responses including *Xoo* and drought, we sought to independently confirm the effects of ABA on *OsWRKY114* expression. To this end, we sprayed 4-week-old rice plants (from the *japonica* cultivar Ilmi) with a 100 µM ABA solution and collected samples at various times for reverse transcription quantitative polymerase chain reaction (RT-qPCR) analysis. The *OsWRKY114* transcript level slightly increased 2 h (hour) after exogenous ABA treatment, but it rapidly reverted to a basal level (Figure 1B). Since OsWRKY114 is involved in disease resistance to *Xoo* [41], we examined whether ABA affects the *OsWRKY114* transcript level during *Xoo* infection. Accordingly, we pretreated the rice cultivar Ilmi with 100 µM ABA or mock (0.02% [*v*/*v*] Tween 20) solution, and then inoculated the plants with the compatible *Xoo* strain KACC10859 three days later, as previously described [29]. Interestingly, *OsWRKY114* transcript level was lower in ABA-pretreated plants than in mock-pretreated plants after *Xoo* inoculation (Figure 1C). This result suggests that OsWRKY114 is negatively regulated by ABA during *Xoo* infection.

### 2.2. OsWRKY114 Alleviates ABA-Dependent Downregulation of Basal Defense Genes during Xoo Infection

**Figure 2:**
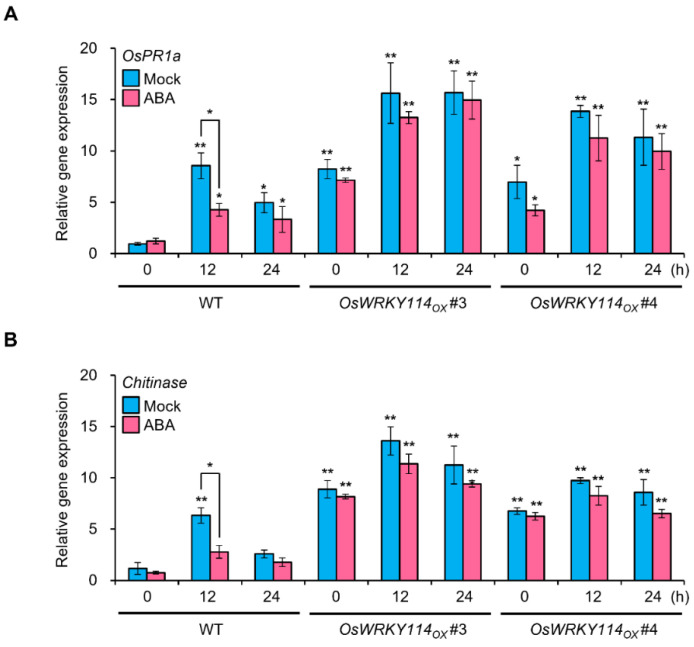
The expression levels of *PR* genes regulated by OsWRKY114 are not suppressed by ABA in *OsWRKY114*-overexpressing plants during *Xanthomonas oryzae* pv. *oryzae* infection. (**A**,**B**) Relative transcript levels for *OsPR1a* (**A**) and *chitinase* (**B**), as determined by RT-qPCR. Four-week-old rice plants were pretreated with 100 µM ABA or mock solution. Three days later, leaves were inoculated with *Xoo* and collected after the indicated incubation times. cDNA was synthesized from total RNA in rice leaves. *OsActin* was used as an internal control. Data are shown as means ± SD. * *p* < 0.05, ** *p* < 0.01, determined by *t*-test relative to the 0 h mock-treated wild-type sample and between the indicated comparing samples.

To confirm that OsWRKY114 is associated with the ABA response during *Xoo* infection, we measured the transcript levels of *PR* genes, such as *OsPR1a* and *chitinase*, whose expression is activated directly by OsWRKY114. Rice plants pretreated with ABA exhibited lower *OsPR1a* and *chitinase* transcript levels at 12 h after compatible *Xoo* inoculation, compared to the mock-treated plants (Figure 2A,B). The expression levels of *OsPR1a* and *chitinase* were reduced according to the same pattern of the *OsWRKY114* transcript level upon ABA treatment. However, *OsPR1a* and *chitinase* transcript levels were not significantly reduced by pretreatment with ABA in transgenic rice plants overexpressing *OsWRKY114* (*OsWRKY114_OX_*) (Figure 2A,B). These results suggest that the inhibition of OsWRKY114 by ABA increases the susceptibility to *Xoo*.

### 2.3. ABA-Induced Susceptibility to Xoo Is Repressed in OsWRKY114-Overexpressing Plants

**Figure 3:**
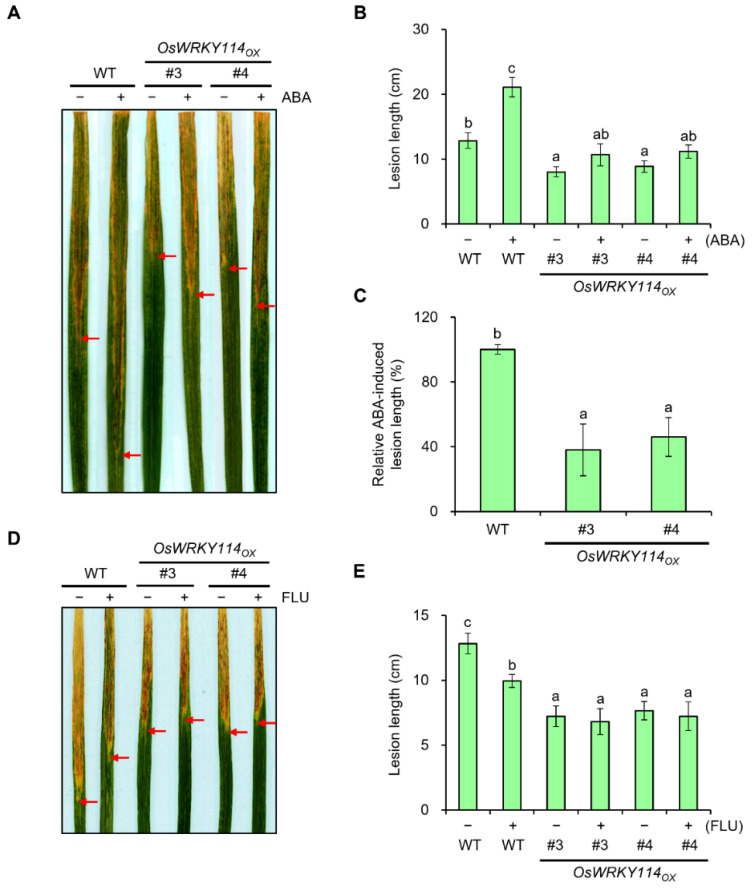
Overexpression of *OsWRKY114* suppresses ABA-induced susceptibility to *Xanthomonas oryzae* pv. *oryzae*. (**A**–**C**) Bacterial blight disease of ABA- or mock-treated rice plants. Four-week-old rice plants were pretreated with 100 µM ABA or mock solution. Three days later, leaves were inoculated with *Xoo*. (**A**) Representative images of lesions captured 14 days after inoculation. (**B**) Lesion length across genotypes and treatments. (**C**) ABA-induced lesion length across genotypes according to the following equation: ABA-induced lesion length (%) = ([Lesion length of ABA-pretreated plants] − [Lesion length of mock-pretreated plants]) × 100. The red arrow indicates the end of the lesion length. Data are shown as means ± SD. Different letters indicate statistical differences according to ANOVA (*p* < 0.05). (**D**,**E**) Bacterial blight disease of FLU- or mock-treated rice plants. Four-week-old rice plants were pretreated with 10 µM FLU or mock solution. Three days later, leaves were inoculated with *Xoo*. (**D**) Representative images of lesions were captured 14 days after inoculation. (**E**) Lesion length across genotypes and treatments. The red arrow indicates the end of the lesion length. Data are shown as means ± SD. Different letters indicate statistical differences according to ANOVA (*p* < 0.05).

To investigate the role of OsWRKY114 in ABA-mediated susceptibility to *Xoo*, we carried out a disease assay in *OsWRKY114_OX_* and wild-type plants. We pretreated 4-week-old rice plants with 100 µM ABA or a mock solution, and inoculated them with *Xoo* 3 days later. The susceptibility of *Xoo* increased in all ABA-treated plants compared to their mock-treated controls (Figure 3A,B). Importantly, the length of ABA-induced lesions was almost 50% less in *OsWRKY114_OX_* plants than in the wild-type plants (Figure 3C). To further confirm the correlation between OsWRKY114 and ABA during *Xoo* infection, we inoculated it in 4-week-old rice plants pretreated with the ABA biosynthesis inhibitor fluridone (FLU). Although the susceptibility of *Xoo* was decreased significantly in FLU-treated wild-type plants compared to their mock-treated controls, it was not in *OsWRKY114_OX_* lines (Figure 3D,E). This result suggests that the *Xoo*-induced ABA biosynthesis is inhibited effectively in *OsWRKY114_OX_* plants. Taken together, these results suggest that OsWRKY114 is a negative regulator of ABA-induced susceptibility to *Xoo*.

### 2.4. The Negative Effect of ABA on SA Defense Mechanism Is Weaker in OsWRKY114-Overexpressing Plants

**Figure 4:**
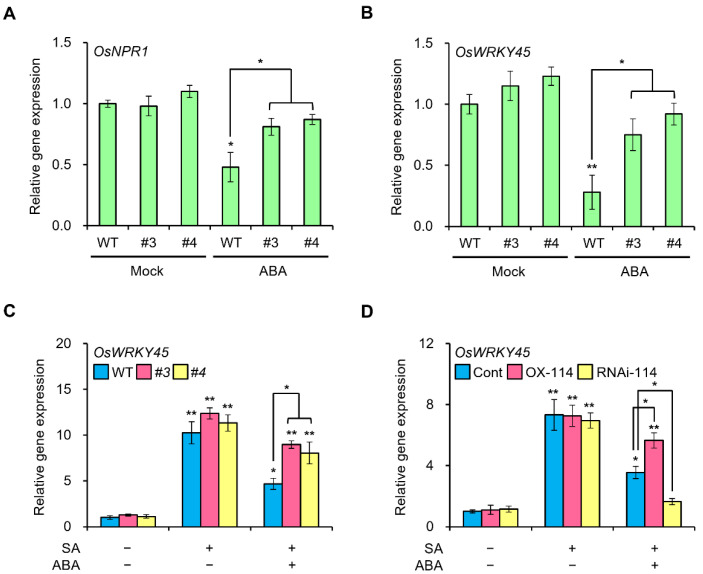
ABA-induced SA signaling suppression is alleviated by OsWRKY114. (**A**,**B**) Relative transcript levels for *OsNPR1* (**A**) and *OsWRKY45* (**B**). Four-week-old rice plants were pretreated with 100 µM ABA or mock solution. Three days later, leaves were inoculated with *Xanthomonas oryzae* pv. *oryzae*. Samples were collected 4 days later for RT-qPCR analysis. *OsActin* was used as an internal control. Data are shown as means ± SD. * *p* < 0.05, ** *p* < 0.01, determined by *t*-test relative to mock-treated wild-type sample and between the indicated comparing samples. (**C**) Gene expression *OsWRKY45* in SA- or SA+ABA-treated *OsWRKY114_OX_* and wild-type plants. A total of 500 µM SA was applied to leaf segments of *OsWRKY114_OX_* and wild-type plants alone or with 50 µM ABA. After 8 h of incubation, total RNA was isolated for RT-qPCR analysis. *OsActin* was used as an internal control. Data are shown as means ± SD. * *p* < 0.05, ** *p* < 0.01, determined by *t*-test relative to non-treated wild-type sample and between the indicated comparing samples. (**D**) Gene expression of *OsWRKY45* in SA- or SA+ABA-treated protoplasts. Rice protoplasts were transfected with or without constructs pEarleyGate104/*OsWRKY114* and pB7GWIWG(II)/*OsWRKY114*-RNAi, respectively, and then 500 µM SA was treated to protoplasts alone or with 50 µM ABA. After 8 h of incubation, total RNA was isolated for RT-qPCR analysis. *OsActin* was used as an internal control. Data are shown as means ± SD. * *p* < 0.05, ** *p* < 0.01, determined by *t*-test relative to non-treated control sample and between the indicated comparing samples.

The expressions of SA marker genes were suppressed at 4 days after compatible *Xoo* inoculation in the ABA-treated rice, but were not suppressed in mock-treated rice [29]. We thus determined the transcript levels of *OsNPR1* and *OsWRKY45* at 4 days after *Xoo* inoculation in *OsWRKY114_OX_* and wild-type plants. ABA pretreatment lowered *OsNPR1* and *OsWRKY45* transcript levels sharply in the wild-type plants; notably, this ABA response was attenuated in *OsWRKY114_OX_* plants after *Xoo* inoculation (Figure 4A,B). However, the two genes were expressed to similar levels across all genotypes in mock-treated plants after *Xoo* inoculation (Figure 4A,B). To clarify the role of OsWRKY114 in SA signaling, we performed the transient protoplast transfection assay to test whether OsWRKY114 might directly regulate the expressions of *OsNPR1* and *OsWRKY45*. We individually introduced the constructs pEarleyGate104/*OsWRKY114* (overexpression) and pB7GWIWG(II)/*OsWRKY114*-RNAi (RNA interference [RNAi]) into rice protoplasts and collected samples after 8 h for RT-qPCR (Supplementary Figure S1A). The transient overexpression of *OsWRKY114* failed to lead to a significant increase in *OsNPR1* and *OsWRKY45* transcript levels, while *OsPR1a* was upregulated as expected (Supplementary Figure S1B). Similarly, silencing of *OsWRKY114* did not affect *OsNPR1* and *OsWRKY45* transcript levels.

To dissect the feature of OsWRKY114 in ABA and SA crosstalk, we treated the phytohormones to *OsWRKY114_OX_* and wild-type plants and measured the gene expression of *OsWRKY45*. The gene expression of *OsWRKY45* was increased by SA but the expression was reduced by SA and ABA co-treatment (Figure 4C). However, the reduction was alleviated in *OsWRKY114_OX_* (Figure 4C). To examine the consequences of *OsWRKY114* overexpression and silencing, we used a protoplast transient transfection system. SA dependently increased *OsWRKY45* was reduced by ABA co-treatment, and the reduction was alleviated in *OsWRKY114*-expressed protoplast, such as in *OsWRKY114_OX_* (Figure 4D), but had a more reduced *OsWRKY45* expression in *OsWRKY114*-RNAi-expressed protoplast (Figure 4D). These results suggest that OsWRKY114 does not directly regulate SA signaling, but indirectly enhances SA-dependent disease resistance by inhibiting ABA response after *Xoo* infection.

### 2.5. Various ABA-Response and ABA-Related Genes Are Downregulated in OsWRKY114-Overexpressing Plants

**Figure 5:**
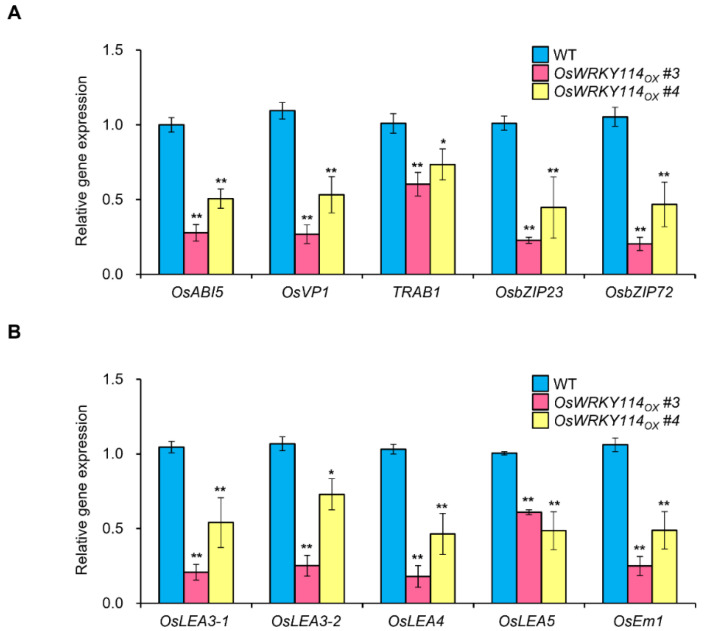
The expressions of various ABA-associated genes are downregulated in *OsWRKY114*-overexpressing plants. (**A**) Relative transcript levels of ABA-response genes in 4-week-old rice plants, as determined by RT-qPCR. cDNA was synthesized from total RNA in rice leaves. *OsActin* was used as an internal control. Data are shown as means ± SD. * *p* < 0.05, ** *p* < 0.01, determined by *t*-test relative to wild-type sample. (**B**) Relative transcript levels of ABA-related genes in 4-week-old rice plants, as determined by RT-qPCR. cDNA was synthesized from total RNA in rice leaves. *OsActin* was used as an internal control. Data are shown as means ± SD. * *p* < 0.05, ** *p* < 0.01, determined by *t*-test relative to wild-type sample.

To explore the regulatory mechanism of OsWRKY114 in the ABA response, we analyzed the transcript levels of various genes associated with ABA by RT-qPCR analysis. We observed that the ABA-response genes *OsABI5* (*ABA-insensitive 5*), *OsVP1* (*Viviparous 1*), *TRAB1* (*Transcription factor responsible for ABA regulation 1*), *OsbZIP23* (*Basic leucine zipper 23*), and *OsbZIP72* are expressed at lower levels in *OsWRKY114_OX_* plants compared to the wild-type plants (Figure 5A). Moreover, we confirmed the repression of ABA response by OsWRKY114, as evidenced by the downregulation of the ABA-related genes *OsLEA3-1* (*Late embryogenesis abundant 3-1*), *OsLEA3-2*, *OsLEA4*, *OsLEA5*, and *OsEm1* (*Embryonic abundant protein 1*) (Figure 5B). These results indicate that OsWRKY114 downregulates the expressions of ABA-associated genes.

### 2.6. ABA Biosynthesis Is Attenuated in OsWRKY114-Overexpressing Plants during Xoo Infection

**Figure 6:**
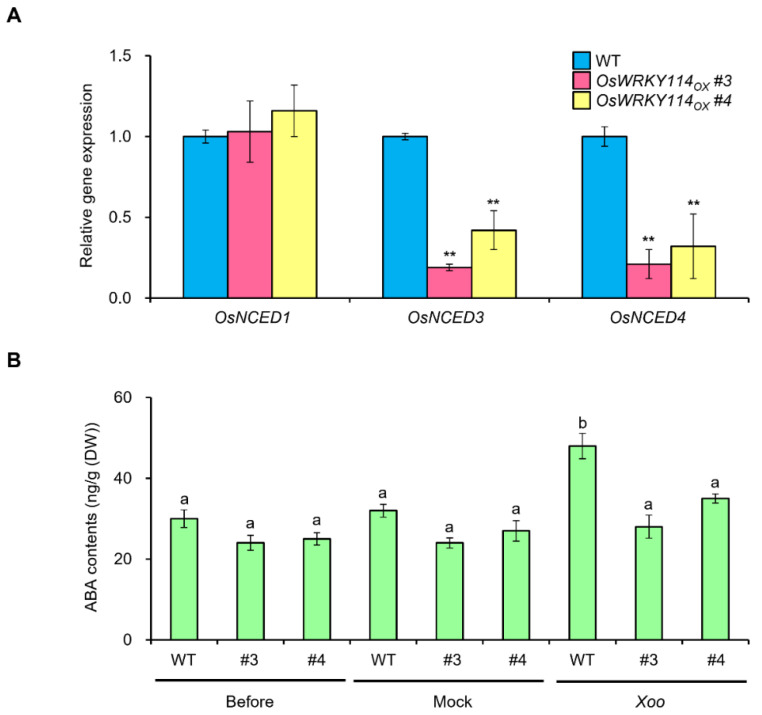
ABA biosynthesis is attenuated in *OsWRKY114*-overexpressing plants after *Xanthomonas oryzae* pv. *oryzae* inoculation. (**A**) Relative *OsNCEDs* transcript levels in 4-week-old *OsWRKY114*-overexpressing and wild-type plants, as determined by RT-qPCR. cDNA was synthesized from total RNA in rice leaves. *OsActin* was used as an internal control. Data are shown as means ± SD. ** *p* < 0.01, determined by *t*-test relative to wild-type sample. (**B**) Analysis of ABA contents in 4-week-old *OsWRKY114*-overexpressing and wild-type plants. Leaves of 4-week-old rice plants were collected or treated with *Xoo* or mock solution. After 8 days, leaves of rice plants treated with *Xoo*- or mock solution were collected to determine endogenous ABA contents. Data are shown as means ± SD. Different letters indicate statistical differences according to ANOVA (*p* < 0.05).

To determine whether *OsWRKY114* is involved in ABA biosynthesis, we monitored the transcript levels of ABA metabolism genes. *OsNCED* genes, such as *OsNCED3* and *OsNCED4,* appeared to be downregulated in *OsWRKY114_OX_* plants relative to the wild-type plants (Figure 6A). OsNCEDs play a critical role in ABA biosynthesis [43,44], prompting us to measure ABA contents in *OsWRKY114_OX_* and wild-type plants. The endogenous ABA level was only slightly lower in *OsWRKY114_OX_* plants compared to wild-type plants (Figure 6B). However, 8 days after *Xoo* inoculation, the endogenous ABA level increased by approximately 50% in wild-type plants relative to mock-treated plants, but it showed no significant differences in *OsWRKY114_OX_* plants (Figure 6B). These results indicate that OsWRKY114 significantly attenuates ABA biosynthesis after *Xoo* infection.

## 3. Discussion

In previous our study, the OsWRKY114 directly activates the promoters of *PR* genes and enhances disease resistance to *Xoo* [41]. Furthermore, interestingly, drought tolerance is reduced in *OsWRKY114_OX_* plants [42]. ABA is the central phytohormone conferring plant tolerance against abiotic stress including drought, whereas it reduces disease resistance to biotrophic pathogens via an antagonistic effect of SA signaling [45]. Therefore, these results suggest that OsWRKY114 may be a negative regulator of ABA. However, the regulation of ABA by OsWRKY114 during *Xoo* infection is not yet established. Here, we demonstrate that OsWRKY114 inhibits ABA-dependent susceptibility during *Xoo* infection.

First, we analyzed the expression pattern of *OsWRKY114* and *PR* genes (i.e., *OsPR1a* and *chitinase*), which revealed that they are downregulated by ABA after *Xoo* inoculation (Figure 1C and Figure 2). To better understand the connection between OsWRKY114 and ABA responses during the rice–*Xoo* interaction, we determined how the *OsWRKY114*-overexpressing transgenic plants respond to ABA and *Xoo* infection. Notably, the greater susceptibility to *Xoo* induced by ABA treatment was less pronounced in *OsWRKY114_OX_* plants, based on relative lesion length (Figure 3C). In addition, we discovered that OsWRKY114 increases SA-dependent disease resistance by inhibiting ABA response after *Xoo* inoculation (Figure 4).

The antagonistic relationship between ABA and SA signaling pathways increases the susceptibility of rice toward *Xoo* [29], but the role of OsWRKY114 in the relationship is not yet identified. The master regulator of SA signaling, OsNPR1, enhances innate immunity to *Xoo* [46,47,48]. OsWRKY45, a key regulator of SA signaling, also improves resistance to *Xoo* [49]. Consistent with the increased resistance to *Xoo* observed in *OsWRKY114_OX_* plants, exogenous application of ABA was accompanied with much lower *OsNPR1* and *OsWRKY45* transcript levels in the wild-type plants after *Xoo* inoculation, while this drop was greatly attenuated in *OsWRKY114_OX_* plants (Figure 4A,B). However, OsWRKY114 did not appear to directly regulate the transcription of *OsNPR1* or *OsWRKY45* (Figure 4). Therefore, we concluded that the higher expression levels of SA marker genes (i.e., *OsNPR1* and *OsWRKY45*) in *OsWRKY114_OX_* plants treated with ABA reflect a suppression of ABA signaling.

Indeed, various ABA-response and ABA-related genes were downregulated in *OsWRKY114_OX_* plants compared to the wild-type plants (Figure 5A,B). Moreover, the ABA biosynthetic genes *OsNCED3* and *OsNCED4* were expressed at lower levels upon *OsWRKY114* overexpression in stable transgenic plants (Figure 6A). NCED enzymes convert 9-*cis*-violaxanthin or 9-*cis*-neoxanthin to the ABA precursor xanthoxin [50], such that a higher expression of *NCED* genes leads to a greater accumulation of ABA in plants [51,52,53]. Especially, OsNCED3 and OsNCED4 were expected to be mainly involved in ABA-induced susceptibility to *Xoo* [29]. Therefore, the downregulation of *OsNCED3* and *OsNCED4* by OsWRKY114 suggests that it plays an important role in ABA biosynthesis during *Xoo* infection. Indeed, *Xoo*-induced ABA accumulation was attenuated in *OsWRKY114_OX_* plants (Figure 6B). These results suggest that OsWRKY114 negatively regulates ABA-induced susceptibility to *Xoo* by repressing ABA biosynthesis. Moreover, in spite of no significant differences of ABA contents between 4-week-old *OsWRKY114_OX_* and wild-type plants (Figure 6B), the expressions of various ABA-associated genes were reduced significantly in *OsWRKY114_OX_* plants compared to wild-type plants (Figure 5). These results reveal that OsWRKY114 can also inhibit ABA response.

**Figure 7:**
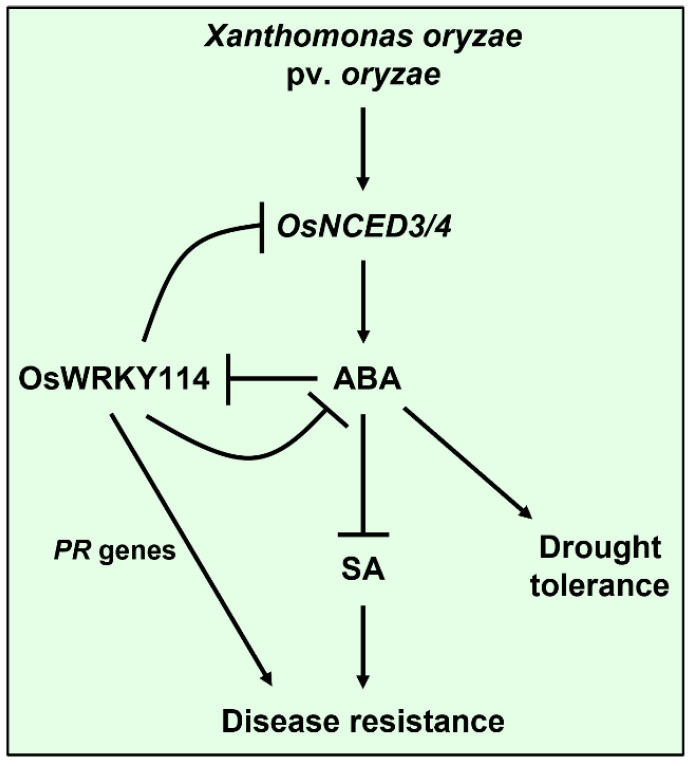
A working model of OsWRKY114 in innate immunity to *Xanthomonas oryzae* pv. *oryzae.* OsWRKY114 enhances disease resistance to *Xoo* through both upregulation of *PR* genes and downregulation of the negative effect of ABA on SA-dependent immunity in rice. However, since ABA is a major phytohormone associated with abiotic stresses, the inhibition of ABA signaling by OsWRKY114 can reduce drought tolerance.

The WRKY Group II transcription factor OsWRKY11 increase plant resistance to both *Xoo* and drought through regulating the expression of biotic and abiotic stress-related genes [54]. However, the WRKY Group III transcription factor OsWRKY45 enhances disease resistance to *Xoo* but reduces drought tolerance in rice [49,55]. Here, we demonstrate the WRKY Group III transcription factor OsWRKY114 increases resistance to *Xoo* with dual regulation of *PR* genes and ABA (Figure 7), while it reduces drought tolerance [42]. The WRKY transcription factors belonging to Group III are known to be the most highly evolved WRKYs [56] and they are involved in mainly innate immunity against various pathogens [57,58,59]. Indeed, OsWRKY45 confers broad-spectrum resistance to various pathogens [49,60,61]. Therefore, OsWRKY114 may also be associated with disease resistance to various pathogens.

In conclusion, the dual function of OsWRKY114 in regulating the expression of *PR* genes and the ABA signaling is important for innate immunity to *Xoo* (Figure 7). OsWRKY114 not only suppressed ABA responses but also reduced pathogen-induced ABA biosynthesis, leading to increased resistance to *Xoo* in rice. However, OsWRKY114 is a transcriptional activator, not repressor [41]. Therefore, a detailed mechanism by which OsWRKY114 negatively regulates ABA response and biosynthesis during *Xoo* infection should be clarified in future studies. Our findings provide valuable information concerning plant–pathogen interactions that may be applicable to plant breeding.

## 4. Materials and Methods

### 4.1. Plant Material and Growth Conditions

The *Oryza sativa* L. *japonica* rice cultivar ‘Ilmi’ was used as the wild-type plant in this study. *OsWRKY114*-overexpressing rice plant lines were previously generated and confirmed [41]. All seeds were surface sterilized with a 5% sodium hypochlorite solution and then rinsed thoroughly with sterilized distilled water. The seeds were germinated in sterilized distilled water for 5 days and then transferred to soil or half-strength Murashige and Skoog (MS) medium and grown under a 16 h light and 8 h dark photoperiod at 28 °C.

### 4.2. Gene Expression Analysis

Expression profiling analysis was performed using Genevestigator (https://genevestigator.com/, (accessed on 11 January 2021)). For RT-qPCR analysis, leaves of rice plants were harvested and frozen in liquid nitrogen and then total RNA was extracted with TRIzol reagent (Invitrogen, Waltham, MA, USA). For each sample, 2 µg total RNA was reverse-transcribed to first-strand cDNA using Superscript III reverse transcriptase (Invitrogen, Waltham, MA, USA) according to the manufacturer’s instructions. RT-qPCR was performed with gene-specific primers (Supplementary Table S1) on the QuantaStudio 3 PCR System (Thermo Fisher Scientific, Waltham, MA, USA) using SYBR Green Master Mix (Enzynomics, Daejeon, Korea) under the following conditions: 40 cycles of denaturation at 95 °C for 10 s, annealing at 58 °C for 15 s, and extension at 72 °C for 30 s. Gene expression was quantified using the comparative Ct method. *OsActin* was used as an internal control to determine gene expression.

### 4.3. Phytohormone and Chemical Treatments

Plants were treated with ABA, SA, and FLU as previously described [29]. Briefly, the ABA (Sigma, St. Louis, MO, USA) concentration was adjusted to 100 µM in 0.02% (*v*/*v*) Tween 20, while the FLU (Sigma, St. Louis, MO, USA) concentration was adjusted to 10 µM. The ABA or FLU solution was then sprayed onto 4-week-old rice plants. Mock-treated plants were sprayed with 0.02% (*v*/*v*) Tween 20. For phytohormone crosstalk experiments, rice leaf segments were incubated for 8 h in the solution containing 500 µM SA (Sigma, St. Louis, MO, USA) with or without 50 µM ABA.

### 4.4. Pathogen Inoculation and Disease Assay

Pathogen inoculation and disease assay were performed as previously described with slight modifications [62]. Briefly, rice plants were grown on soil for 4 weeks, and then sprayed with ABA or mock solution. Three days later, the sprayed plants were inoculated with the compatible *Xoo* strain KACC10859 by the leaf-clipping method. The length of disease lesions was measured at 14 days post-inoculation. The length of the ABA-induced lesions was calculated according to the following equation: ABA-induced lesion length (%) = ([Lesion length of ABA-pretreated plants] − [Lesion length of mock-pretreated plants]) × 100.

### 4.5. Transient Gene Expression Assay in Protoplasts

pEarleyGate104/*OsWRKY114* and pB7GWIWG(II)/*OsWRKY114*-RNAi constructs were previously generated [41]. Transient protoplast transfection and phytohormone treatment were performed as previously described [63] with a slight modification. Briefly, rice seedlings were grown on half-strength MS medium for 2 weeks. Protoplasts were isolated and the constructs were individually transfected by the polyethylene glycol (PEG)-mediated transfection method. The transfected protoplasts were incubated in WI solution at 28 °C. For phytohormone treatment, 500 mM SA with or without 50 µM ABA was added to the solution after 1 h of incubation. After 8 h incubation, the protoplasts were collected for total RNA extraction.

### 4.6. Analysis of ABA Contents

ABA contents were determined as previously described [64]. Briefly, around 5 g of 4-week-old rice leaves were ground to powder with a mortar and pestle in liquid nitrogen. The powder was homogenized with 20 mL of 80% (*v*/*v*) methanol for 30 min on ice. The samples were filtered onto two layers of Miracloth and the solution evaporated under vacuum. The residue was dissolved in 0.5 M phosphate buffer (pH 8.0) with gentle stirring for 30 min. After centrifugation, the supernatant was discarded and the pellet was washed with 20 mL of mineral spirit. The pH was adjusted to 2.8 with 12 M hydrochloric acid. The samples were centrifuged again and resuspended in 10 mL ethyl acetate. This step was repeated three more times and the supernatants pooled. The extracts were lyophilized and dissolved in 5 mL of 0.5 M phosphate buffer (pH 8.0). The samples were purified on a Sephadex^®^ G-10 column (Sigma, St. Louis, MO, USA). The eluates were lyophilized and dissolved in 1 mL acetonitrile and analyzed by HPLC (Chromaster, Hitachi, Japan) (+)-ABA was purchased from Sigma (Sigma, St. Louis, MO, USA) and used as standard.

### 4.7. Statistical Analysis

All experiments were independently conducted at least three times, and the average values from the independent experiments were presented. The data were analyzed by *t*-test or ANOVA. Asterisks denote significant differences (* *p* < 0.05, ** *p* < 0.01) and different letters indicate statistical differences (*p* < 0.05).

## Data Availability

The data presented in this study are available in the article or the Supplementary Materials.

## Supplementary Materials

The following are available online at https://www.mdpi.com/article/10.3390/ijms23158825/s1.
