# Supplementary material for: OsWRKY114 Inhibits ABA-Induced Susceptibility to Xanthomonas oryzae pv. oryzae in Rice
Source: Int J Mol Sci. 2022 Aug 8;23(15):8825. doi: 10.3390/ijms23158825 (PMC9369203; doi:10.3390/ijms23158825)
Supplement: Supplementary file 1 [file ijms-23-08825-s001.zip › ijms-1845558-supplementary.pdf]

A

B

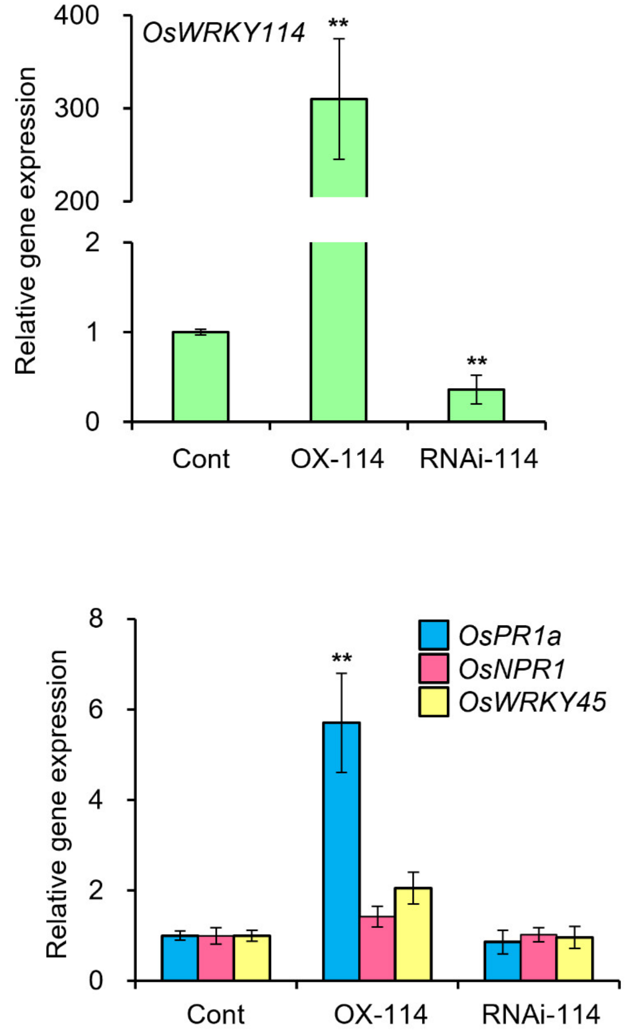

Figure S1. Relative transcript levels of SA marker genes in protoplast. (**A, B**) Relative transcript levels of *OsWRKY114* (**A**) and SA marker genes such as *OsNPR1* and *OsWRKY45* (**B**) in protoplast. Rice protoplasts were transfected with or without constructs pEarleyGate104/*OsWRKY114* and pB7GWIWG(II)/*OsWRKY114*-RNAi, respectively. After an incubation of 8 h, total RNA was extracted for RT-qPCR analysis. *OsActin* was used as an internal control. Data are shown as means ± SD. ** $p < 0.01$, by *t*-test relative to control sample.

Gene	Primer sequence	Purpose
OsWRKY114	F: CACCCATGTTCCAAGTGACA	qRT-PCR
	R: ATCGTCAGGGTGACCATTTG	qRT-PCR
OsPR1a	F: GGAAGTACGGCGAGAACATC	qRT-PCR
	R: TGGTCGTACCACTGCTTCTC	qRT-PCR
Chitinase	F: CGCCCTAAGATAGAGTAACATCG	qRT-PCR
	R: CAAGAGCAACAAACAGTGGC	qRT-PCR
OsABI5	F: GCAGGAGGAAGCGGTTTATG	qRT-PCR
	R: AGTGTTACCTGTCGGTCGTT	qRT-PCR
OsVP1	F: ACATAGACCAGCTCCTCGAC	qRT-PCR
	R: GGACATGGCGTCCTCTATCA	qRT-PCR
TRAB1	F: CCGGCTGTCAACAACAGCTC	qRT-PCR
	R: GAAACCCATCGCACCAGCAG	qRT-PCR
OsbZIP23	F: GAGATCACGCTGGAGGAGTT	qRT-PCR
	R: ACCATCGGAGGGAACACATT	qRT-PCR
OsbZIP72	F: TGTATTCGCTGACGTTCGAC	qRT-PCR
	R: GAGCAGCTCGTCCATGTTC	qRT-PCR
OsLEA3-1	F: ATACCAAGGAGGCGACGAAG	qRT-PCR
	R: GTGCTGGAGGTCTTGTCCTT	qRT-PCR
OsLEA3-2	F: AAGAACAAGCTGGGCGAGTA	qRT-PCR
	R: CTTGAACTCCGTCGCCTTC	qRT-PCR
OsLEA4	F: CCGACGAGAAGAAGGAGGTG	qRT-PCR
	R: TTATGGAGCCTGTGCTGGAT	qRT-PCR
OsLEA5	F: GCTCACCTACACCCTCAAGT	qRT-PCR
	R: GATCTTGGCCGGTATCTCCA	qRT-PCR
OsEm1	F: AGACGAGGAAGGAGCAGAT	qRT-PCR
	R: GGACTTGGTCTTGTACTTG	qRT-PCR
OsNCED1	F: CTACATCCCTCCTGCTGCTT	qRT-PCR
	R: CTACCAACTGCTCGTCCTCT	qRT-PCR
OsNCED3	F: TTCGCCATCACCGAGAACTA	qRT-PCR
	R: GAGCATCTCCTGGAGCTTGA	qRT-PCR
OsNCED4	F: GTCCAAGCCGTACCTCAAGT	qRT-PCR
	R: TCCTGGAGCTTGAACACGAT	qRT-PCR
OsNPR1	F: CACGCCTAAGCCTCGGATTA	qRT-PCR
	R: TCAGTGAGCAGCATCCTGACTAG	qRT-PCR
OsWRKY45	F: GGACGCAGCAATCGTCCGGG	qRT-PCR
	R: CGGAAGTAGGCCTTTGGGTGC	qRT-PCR
OsActin	F: CATTGGTGCTGAGCGTTTCC	qRT-PCR
	R: CTCCTTGCTCATCCTGTCAGC	qRT-PCR

Table S1. Sequence of primers used in this study.
